# Traumatic Brain Injury‐associated gene network‐based prediction and population‐based validation identifies doxycycline as a repurposable medicine for Alzheimer’s Disease

**DOI:** 10.1002/alz70859_103656

**Published:** 2025-12-25

**Authors:** Marina Bykova, Reina Tonegawa Kuji, Ehud Karavani, Michael Danziger, Michal Rosen‐Zvi, Andrew A. Pieper, Feixiong Cheng

**Affiliations:** ^1^ Lerner Research Institute, Cleveland Clinic Foundation, Cleveland, OH USA; ^2^ Biological, Geological, and Environmental Sciences, Cleveland State University, Cleveland, OH USA; ^3^ Lerner Research Institute, Cleveland, OH USA; ^4^ IBM Research, Haifa, Haifa Israel; ^5^ Brain Health Medicines Center, Harrington Discovery Institute, University Hospitals Cleveland Medical Center, Cleveland, OH USA; ^6^ Louis Stokes VA Medical Center, Cleveland, OH USA; ^7^ Case Western Reserve University, Cleveland, OH USA; ^8^ Louis Stokes Cleveland VA Medical Center, Cleveland, OH USA; ^9^ Cleveland Clinic Genome Center, Cleveland, OH USA; ^10^ Cleveland Clinic, Cleveland, OH USA

## Abstract

**Background:**

Traumatic Brain Injury (TBI) is one of the most common causes of Alzheimer’s disease (AD). However, few effective treatments exist for AD, and even fewer for TBI. Previously demonstrated network‐medicine techniques using gene set data and electronic health records analysis can be used for identification of drug repurposing candidates for the prevention of AD within elderly and post‐TBI patient populations.

**Method:**

We used a network‐based approach to assess gene set connectedness, evaluating shortest path lengths between disease‐associated genes in a protein‐protein interactome (PPI) network via permutation tests with 1,000 random subnetworks. The application of the algorithm links together a curated human interactome network to a drug‐target network, to create ranked lists of repurposable drug candidates. We further tested top network‐predicted drugs using large‐scale patient insurance claims data from the MarketScan^®^ database.

**Result:**

We collected 69 TBI and 144 previously published AD genes and processed them separately using the Python‐platformed algorithm, to generate four ranked drug lists. Candidates were selected to remove drugs that are over‐the‐counter, non‐FDA approved, used for chemotherapy, have severe side effects, or associated with a higher dementia risk. Drugs were later filtered on a Z‐score threshold (Z‐score ≤ ‐2) and prioritized based on presence in multiple post‐threshold candidate lists and/or use in AD‐related research, before evaluation against patient cohorts within the MarketScan^®^ database. Candidates were examined over a 3‐year observation period in patients over the age of 70 for AD diagnosis incidence. We identified that doxycycline prescription was associated with a lowered risk of developing AD compared to patients prescribed Anatomical Therapeutic Chemical (ATC) codes anti‐infectives (risk ratio (RR) = 0.92, adjusted 95% confidence interval (CI) 0.87‐0.96, *p*‐value = 0.002) and antibiotics (RR = 0.92, adjusted 95% CI 0.87‐0.97, *p*‐value = 0.002). Possible mechanisms of action include effects on microglial activity and blood‐brain barrier integrity.

**Conclusion:**

We demonstrated that combining network‐based predictions and large real‐world patient data observations can be used to identify potential repurposable drugs for AD based on combined AD‐TBI associated genes within the human protein interactome. This has led to identifying doxycycline as a candidate treatment for preventing AD.

